# MICA-129 Dimorphism and Soluble MICA Are Associated With the Progression of Multiple Myeloma

**DOI:** 10.3389/fimmu.2018.00926

**Published:** 2018-05-01

**Authors:** Alessandra Zingoni, Elisabetta Vulpis, Francesca Cecere, Maria G. Amendola, Daniel Fuerst, Taron Saribekyan, Adnane Achour, Tatyana Sandalova, Ilaria Nardone, Agnese Peri, Alessandra Soriani, Cinzia Fionda, Elena Mariggiò, Maria T. Petrucci, Maria R. Ricciardi, Joannis Mytilineos, Marco Cippitelli, Cristina Cerboni, Angela Santoni

**Affiliations:** ^1^Department of Molecular Medicine, “Sapienza” University of Rome, Rome, Italy; ^2^Istituto Pasteur Italia-Cenci Bolognetti Fondazione, Rome, Italy; ^3^German Red Cross Blood Donor Services, Baden-Wuerttemberg-Hessia, Ulm, Germany; ^4^Science for Life Laboratory, Department of Medicine Solna, Karolinska Institutet, Stockholm, Sweden; ^5^Division of Infectious Diseases, Karolinska University Hospital, Stockholm, Sweden; ^6^Department of Cellular Biotechnologies and Hematology, “Sapienza” University of Rome, Rome, Italy; ^7^Department of Clinical and Molecular Medicine, “Sapienza” University of Rome, Rome, Italy

**Keywords:** multiple myeloma, natural killer cells, NKG2D receptor, MICA polymorphism, predictive biomarker

## Abstract

Natural killer (NK) cells are immune innate effectors playing a pivotal role in the immunosurveillance of multiple myeloma (MM) since they are able to directly recognize and kill MM cells. In this regard, among activating receptors expressed by NK cells, NKG2D represents an important receptor for the recognition of MM cells, being its ligands expressed by tumor cells, and being able to trigger NK cell cytotoxicity. The MHC class I-related molecule A (MICA) is one of the NKG2D ligands; it is encoded by highly polymorphic genes and exists as membrane-bound and soluble isoforms. Soluble MICA (sMICA) is overexpressed in the serum of MM patients, and its levels correlate with tumor progression. Interestingly, a methionine (Met) to valine (Val) substitution at position 129 of the α2 heavy chain domain classifies the MICA alleles into strong (*MICA-129Met*) and weak (*MICA-129Val*) binders to NKG2D receptor. We addressed whether the genetic polymorphisms in the MICA-129 alleles could affect MICA release during MM progression. The frequencies of *Val/Val, Val/Met*, and *Met/Met* MICA-129 genotypes in a cohort of 137 MM patients were 36, 43, and 22%, respectively. Interestingly, patients characterized by a *Val/Val* genotype exhibited the highest levels of sMICA in the sera. In addition, analysis of the frequencies of MICA-129 genotypes among different MM disease states revealed that *Val/Val* patients had a significant higher frequency of relapse. Interestingly, NKG2D was downmodulated in NK cells derived from *MICA-129Met/Met* MM patients. Results obtained by structural modeling analysis suggested that the Met to Val dimorphism could affect the capacity of MICA to form an optimal template for NKG2D recognition. In conclusion, our findings indicate that the *MICA-129Val/Val* variant is associated with significantly higher levels of sMICA and the progression of MM, strongly suggesting that the usage of soluble MICA as prognostic marker has to be definitely combined with the patient MICA genotype.

## Introduction

Natural killer (NK) cells represent innate immune effectors playing a pivotal role in tumor surveillance. NK cell activation is regulated by a delicate balance between activating and inhibitory signals, with the latter being primarily transduced by receptors for MHC class I molecules (KIRs, CD94/NKG2A). Recognition of abnormal self on tumor cells triggers a number of non-MHC class I-restricted activating receptors, such as NK group 2D (NKG2D), DNAX accessory molecule-1 (CD226), and the natural cytotoxicity receptors (1).

NKG2D is an activating receptor expressed on the surface of NK cells, CD8^+^ T cells, and subsets of CD4^+^ T cells, invariant NKT cells (iNKT), and γδ T cells (1). NKG2D recognizes two families of ligands in humans: the MHC class I chain-related protein A/B (MICA/B) and the UL16-binding proteins (ULBP1-6) (1). In general, healthy adult tissues do not express NKG2D ligands on the cell surface, but the expression levels of these molecules can be significantly induced by various physiological and pathological “stress” circumstances, including infection by different pathogens (1), cell division (2), and neoplastic transformation (3). Among all known NKG2D ligands, *MICA* is the most polymorphic non classical class I gene, with 104 alleles identified to date (http://www.ebi.ac.uk/imgt/hla/, release 3.25.0). Some MICA polymorphisms have raised a great interest since they can affect MICA biology. For instance, the MICA*008 allele (rs67841474) contains a guanine (G) insertion that causes a premature stop codon that, in turn, crops 10 amino acids of the transmembrane domain as well as the cytoplasmic tail. In contrast to other MICA alleles that are shed as truncated soluble species after metalloproteinase-mediated cleavage, it is released from cells associated to exosomes (4). In addition, the single-nucleotide polymorphism causing a valine (Val) to methionine (Met) modification at position 129 of the α2 heavy chain domain classifies these MICA alleles into high-affinity (*MICA-129Met*) and low-affinity (*MICA-129Val*) binders to NKG2D receptor (5). It has also been recently reported that *MICA-129Met*, characterized by stronger and faster NKG2D signaling, is able to trigger relatively higher NK cell cytotoxicity and IFNγ release accompanied by rapid downregulation of NKG2D (6). Significant differences in binding affinities of MICA alleles for NKG2D could have different effects on NK cell activation, in particular under conditions of suboptimal MICA expression.

Multiple myeloma (MM) is a clonal B cell malignancy characterized by the expansion of plasma cells (PCs) in the bone marrow (BM) and is still an incurable disease with a median survival of few years. Its prognosis has been improved by the use of autologous hematopoietic stem cell transplantation (7) and new immunochemotherapeutic approaches (8–10). NK cells play a pivotal role in MM immunosurveillance by exerting direct cytotoxic effects through a number of activating receptors, including NKG2D (11, 12). However, several mechanisms have been identified that permit the escape of tumors bearing NKG2D ligands, including their release by tumor cells through proteolytic cleavage (13–17) or exosome secretion (4). Furthermore, it has been demonstrated that MICA is transferred to NK cells upon target conjugation and that this transfer is directly linked to molecular interactions between NKG2D and MICA, following accumulation of the ligand at the immunological synapse (18). Soluble MICA has been identified as an independent prognostic factor for the overall survival and progression-free survival of MM patients (19). In addition, endogenous anti-MICA antibodies and ligand shedding are critical determinants of host immunity during MM progression (20). It is, however, unknown whether functionally relevant polymorphisms of the *MICA* gene may also contribute to disease progression.

The aim of this study was to investigate the association of MICA genetic polymorphisms and MICA sera levels with progression of MM. Interestingly, our findings indicate that the *MICA-129Met/Val* dimorphism is associated with: (i) differential expression of both soluble and cell-surface MICA, (ii) expression levels of NKG2D on *ex vivo* NK cells isolated from the BM and peripheral blood (PBL) of MM patients, and (iii) the disease state.

## Results

### *MICA-129Val* Allele Is Associated With Higher Amount of Soluble MICA in the Sera of MM Patients

**Figure 1:**
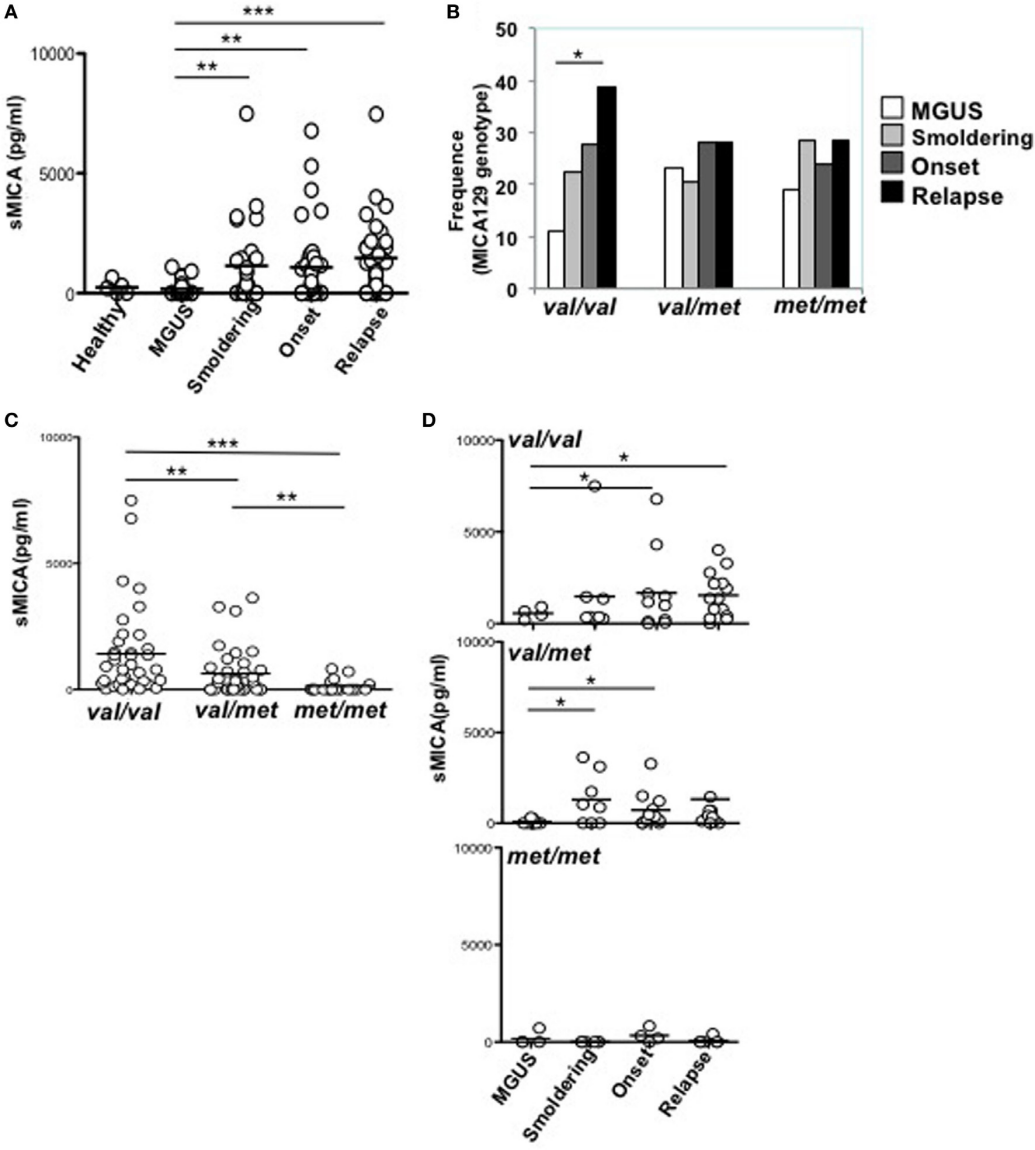
*MICA-129Val* allele is associated with higher amount of sMICA in the sera of multiple myeloma (MM) patients. **(A)** Sera derived from healthy donors, MGUS, and MM patients at different state disease were analyzed for the presence of soluble MICA through a specific enzyme-linked immunosorbent assay. Total number of patients, 97 (healthy, *n* = 5, MGUS, *n* = 16, smoldering, *n* = 22, onset, *n* = 26, relapse, *n* = 32). **(B)** Frequency distribution of MICA-129 genotypes among different MM disease states. χ^2^ test with *n −* 1 degrees of freedom was performed. **(C,D)** sMICA is associated with the presence of the *MICA-129* valine (*Val*) allele in MM patients. Total number of patients analyzed, *n* = 91 (Val/Val, *n* = 37; Val/Met, *n* = 36; Met/Met, *n* = 18).

**Figure 2:**
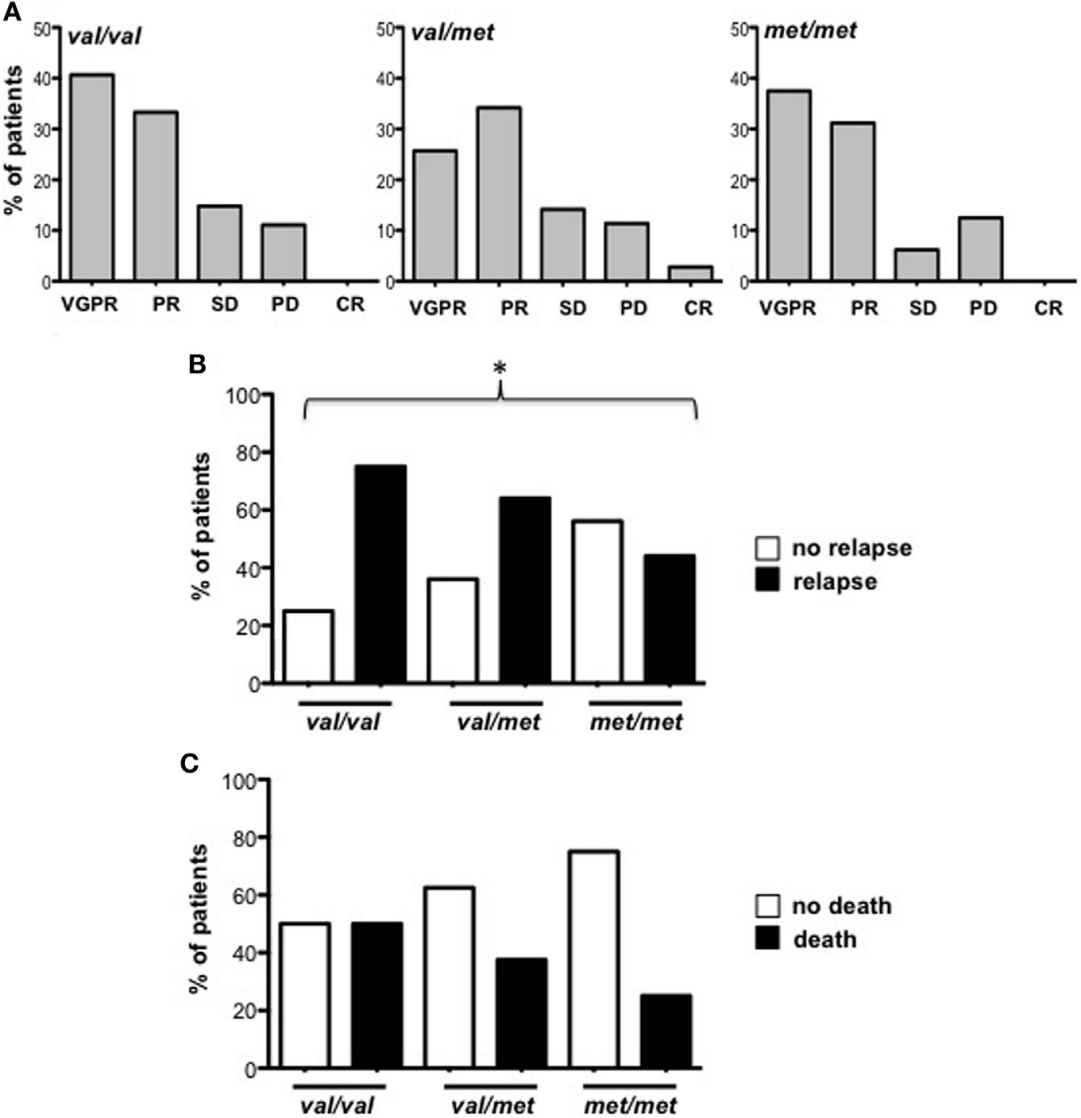
Patient outcome related to MICA-129 genotype. **(A)** Response to therapeutic treatment of multiple myeloma (MM) patients (Onset and Relapse). VGPR, very good partial response; PR, partial response; SD, stable disease; PD, partial disease, CR, complete remission. **(B)** Frequency of relapse development in MM patients after chemotherapy. χ^2^ test, *p* = 0.0413. **(C)** Frequency of deceased patients in MM patients after chemotherapy. Total patients, *n* = 74; Val/Val, *n* = 26; Val/Met, *n* = 32; Met/Met, *n* = 16. Abbreviations: Val, valine; Met, methionine.

Soluble MICA has been proposed as a prognostic marker in MM since its levels correlate with tumor progression (19). However, the generation of soluble MICA can be affected by polymorphisms, regulating cell-surface expression, altering the efficacy of cleavage, and favoring MICA recruitment into exosome-like vesicles (4, 16, 21, 22). At first, we investigated whether soluble NKG2D ligands other than MICA in the sera derived from a cohort of MM patients at different disease states, namely MGUS (monoclonal gammopathy of undetermined significance), smoldering, onset, and relapse, were associated with MM progression. As shown in Figure 1A, we established that only soluble MICA but not other soluble NKG2D ligands including MICB and ULBP1-3 (data not shown) were associated to MM progression. Since we confirmed the importance of soluble MICA, we further explored whether MICA polymorphism could affect the amount of soluble MICA as well as MM progression. Therefore, MICA genotype was examined by isolating PBMCs DNA from a cohort of 137 MM patients at different disease states (Figure S1A in Supplementary Material). We also identified the sequence of a new MICA allele and the name MICA*085 has been officially assigned by the WHO Nomenclature Committee for factors of the HLA System. MICA alleles were further classified in three subgroups, *MICA-129Val/Val, MICA-129Val/Met*, and *MICA-129Met/Met* (Tables 1 and 2). Similarly to other studies (6, 23–25), the frequencies of *MICA-129Val/Val, MICA-129Val/Met*, and *MICA-129Met/Met* genotypes were 36, 42, and 22%, respectively (Figure S1B in Supplementary Material). Remarkably, the analysis of *MICA-129* genotype frequencies among different MM states revealed that *MICA-129Val/Val* patients displayed a significantly higher percentage of relapse (Figure 1B; Figure S1C in Supplementary Material). In contrast, the frequencies of *MICA-129Val/Met* and *MICA-129Met/Met* genotypes were similar throughout all the different disease states (Figure 1B). Interestingly, MM patients characterized by the *MICA-129Val/Val* genotype also exhibited the highest levels of soluble MICA in the sera (Figure 1C). Consequently, correlation of soluble MICA with MM progression was observed only in the presence of the *MICA-129Val* allele (Figure 1D). We further explored the outcome of patients in response to the therapy among different MICA-129 genotypes (Figures 2A–C). As shown in Figure 2A, a similar response to chemotherapy was observed among the three genotypes. Interestingly, we observed that the highest frequency of relapse was developed by *MICA-129Val/Val* patients also after chemotherapeutic treatment (Figure 2B) suggesting that MICA polymorphism impacts on MM relapse.

**Table 1:** MICA genotype and 129 polymorphism in patients at different disease state.

MGUS patients MICA genotype-129 polymorphism		Smoldering patients MICA genotype-129 polymorphism		Onset patients MICA genotype-129 polymorphism		Relapse patients MICA genotype-129 polymorphism	
002:01/018:01	Met/Met	002:01/018:01	Met/Met	009:01/018:01	Val/Met	085/085	Val/Val
008:01/016	Val/Val	018:01/018:01	Met/Met	002:01/18:01	Met/Met	002:01/008:01	Met/Val
004/012:01	Val/Met	008:01/008:01	Val/Val	004/009:02	Val/Val	002:01/016	Met/Val
016/019	Val/Val	017/019	Met/Val	004/008:01	Val/Val	004/016	Val/Val
008:01/018:01	Val/Met	008:01/011	Val/Met	002:01/010:01	Met/Val	009:01/009:01	Val/Val
002:01/018:01	Met/Met	002:01/018:01	Met/Met	004/008:01	Val/Val	002:01/004	Met/Val
001/018:01	Met/Met	006/008:01	Val/Val	010:01/017	Val/Met	002:01/018:01	Met/Met
006/008:01	Val/Val	009:01/018:01	Val/Met	008:01/018	Val/Met	009:01/009:01	Val/Val
002:01/011	Met/Met	001/018:01	Met/Met	008:01/008:01	Val/Val	016/019	Val/Val
009:01/018:01	Val/Met	002:01/018:01	Met/Met	027/027	Val/Val	008:01/009:01	Val/Val
010:01/018:01	Val/Met	009:01/009:01	Val/Val	008:01/010:01	Val/Val	008:01/009:01	Val/Val
008:01/011	Val/Met	010:01/012:01	Val/Met	004/004	Val/Val	008:01/019	Val/Val
009:01/018:01	Val/Met	004/008:01	Val/Val	004/008:01	Val/Val	009:01/016	Val/Val
004/018:01	Met/Val	008.01/018:01	Val/Met	008:01/018:01	Val/Met	008:01/009:01	Val/Val
002:01/008:01	Val/Met	004/008:01	Val/Val	004/016	Val/Val	002:01/004	Met/Val
004/008:01	Val/Val	002:01/047	Met/Met	002:01/018:01	Met/Met	009:02/018:01	Val/Met
002:01/008:01	Met/Val	009:01/009:02	Val/Val	008:01/018:01	Val/Met	004/009:02	Val/Val
004/011	Val/Met	008:01/009:01	Val/Val	007:01/016	Met/Val	008:01/016	Val/Val
002:01/017	Met/Met	008:01/012:01	Val/Met	004/008:01	Val/Val	001:01/018	Met/Met
002:01/008:01	Met/Val	011/018:01	Met/Met	012:01/018	Met/Met	001/002:01	Met/Met
008:01/008:01	Val/Val	006/008:01	Val/Val	002:01/004	Met/Val	002:01/007:01	Met/Met
008:01/012:01	Val/Met	008:01/010:01	Val/Val	006/009:01	Val/Val	008:01/018:01	Val/Met
002:01/009:02	Met/Val	002:01/004	Met/Val	008:01/018:01	Val/Met	009:01/018:01	Val/Met
011/012:01	Met/Met	002:01/009:01	Met/Val	008:01/018:01	Val/Met	009:01/016	Val/Val
009:01/016	Val/Val	002:01/018:01	Met/Met	002:01/002:01	Met/Met	002:01/018:01	Met/Met
004/016	Val/Val	002:01/008:01	Met/Val	002:01/012:01	Met/Met	004/008:01	Val/Val
002:01/018:01	Met/Met	009:02/018:01	Val/Met	002:01/016	Met/Val	007:01/008:01	Met/Val
002:01/007:01	Met/Met			018:01/027	Met/Val	008:01/010:01	Val/Val
011/047	Met/Met			001/008:01	Met/Val	002:01/007:01	Met/Met
008:01/016	Val/Val			002:01/018:01	Met/Met	009:01/016	Val/Val
004/011	Val/Met			007:01/008:01	Met/Val	001/016	Met/Val
				002:01/008:01	Met/Val	002:01/016	Met/Val
				009:01/009:01	Val/Val	008:01/016	Val/Val
				001/004	Met/Val	002:01/016	Met/Val
				006/018:01	Val/Met	008:01/010:01	Val/Val
				007:01/009:01	Met/Val	009:01/016	Val/Val
				002:01/018:01	Met/Met	008:01/009:01	Val/Val
				004/018:01	Val/Met	002:01/010:01	Met/Val
						018:01/018:01	Met/Met
						002:01/008:01	Met/Val
						008:01/017	Val/Met
						009:01/018:01	Val/Met
						002:01/008:01	Met/Val

*Val, valine; Met, methionine*.

**Table 2:** Patient characteristics.

Patients characteristics	Val/Val	Val/Met	Met/Met
Gender			
Male	25	31	13
Female	24	26	18
Age mean (range)	70 (47–83)	65(41–83)	62 (41–87)
% of PCs mean (range)	MGUS 3 (1–10) Smoldering 16 (5–38) Onset 29 (11–45) Relapse 27 (4–90)	MGUS 4 (1–9) Smoldering 18 (4–40) Onset 36 (7–90) Relapse 29 (5–58)	MGUS 3 (1–10) Smoldering 26 (13–58) Onset 29 (2–52) Relapse 33 (4–54)

*PCs, plasma cells; Val, valine; Met, methionine*.

**Figure 3:**
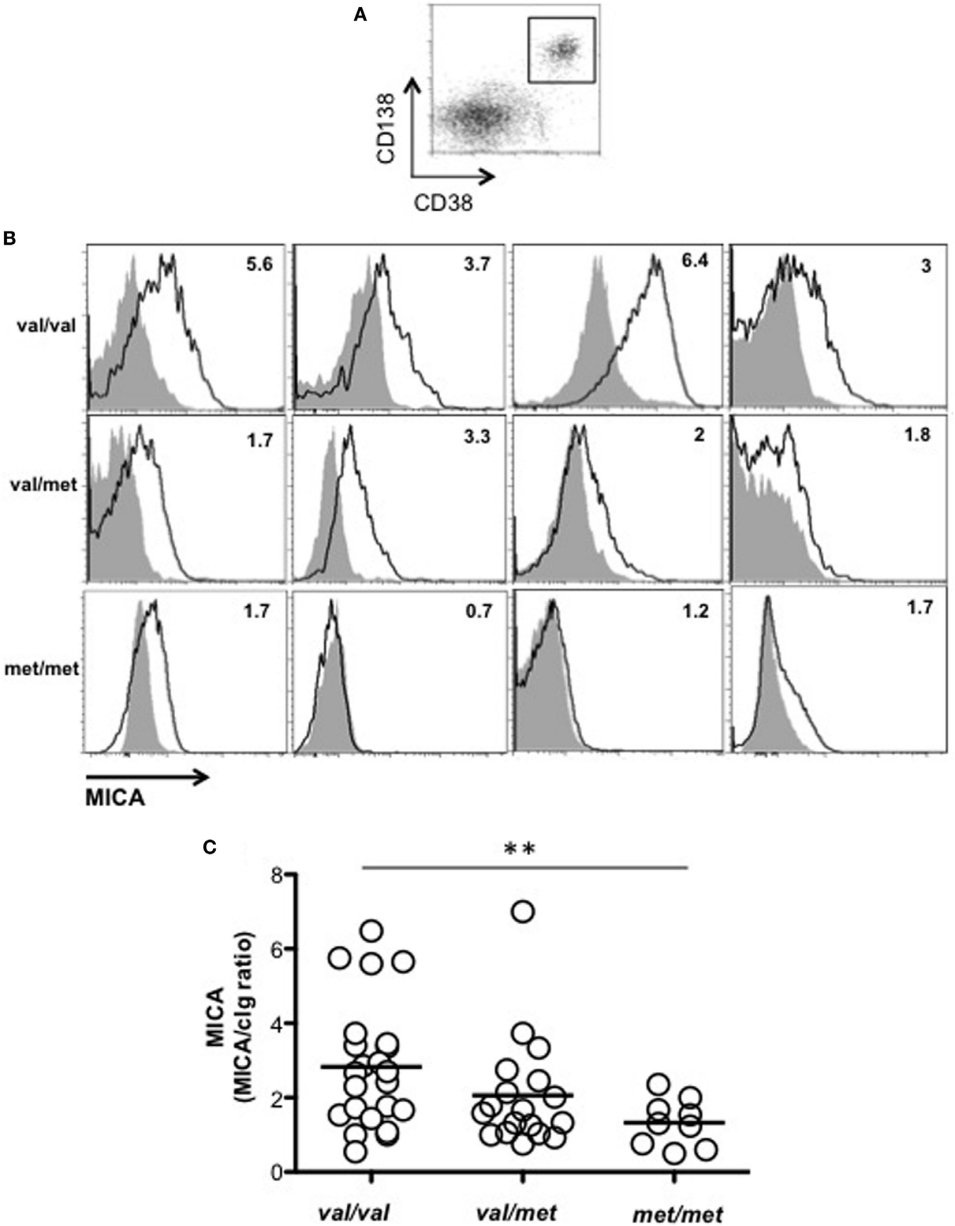
MICA cell-surface expression on malignant plasma cells (PCs) resulted higher in MICA-129Val/Val patients. Total cells derived from the bone marrow (BM) of patients at different disease states were stained with isotypic cIg or anti-MICA together with anti-CD38 and anti-CD138 mAbs. **(A)** MICA expression was analyzed by gating CD38^+^/CD138^+^ cells. **(B)** Representative histograms of different patients are reported. Values indicated in each histogram represent the ratio between the MFI value of MICA divided by the MFI value of the isotypic cIg. Solid gray histogram: cIg; black line: MICA. **(C)** Total number of patients analyzed, *n* = 50 (Val/Val = 23, Val/Met *n* = 18, Met/Met = 9) that were smoldering (*n* = 10), onset (*n* = 19), and relapse (*n* = 21). Abbreviations: Val, valine; Met, methionine.

We asked whether MICA cell-surface expression levels on primary malignant PCs isolated from patients could be related to the MICA genotype. As shown in Figures 3A–C, MICA expression on malignant PCs, was significantly higher in *MICA-129Val/Val* MM patients compared to *MICA-129Met/Met* MM patients, thus suggesting that the increased amount of soluble MICA in the sera of *MICA-129Val/Val* patients could be related to an higher expression of this allelic variant. Finally, we further classified MICA alleles into MICA short and long, based on the presence of the truncated MICA*008 allelic variant, but no differences regarding soluble MICA serum levels and the correlation with the disease state were found (data not shown).

### *MICA-129Met* Allele Is Associated With an Increased NKG2D Downmodulation on NK Cells Isolated From Patients

**Figure 4:**
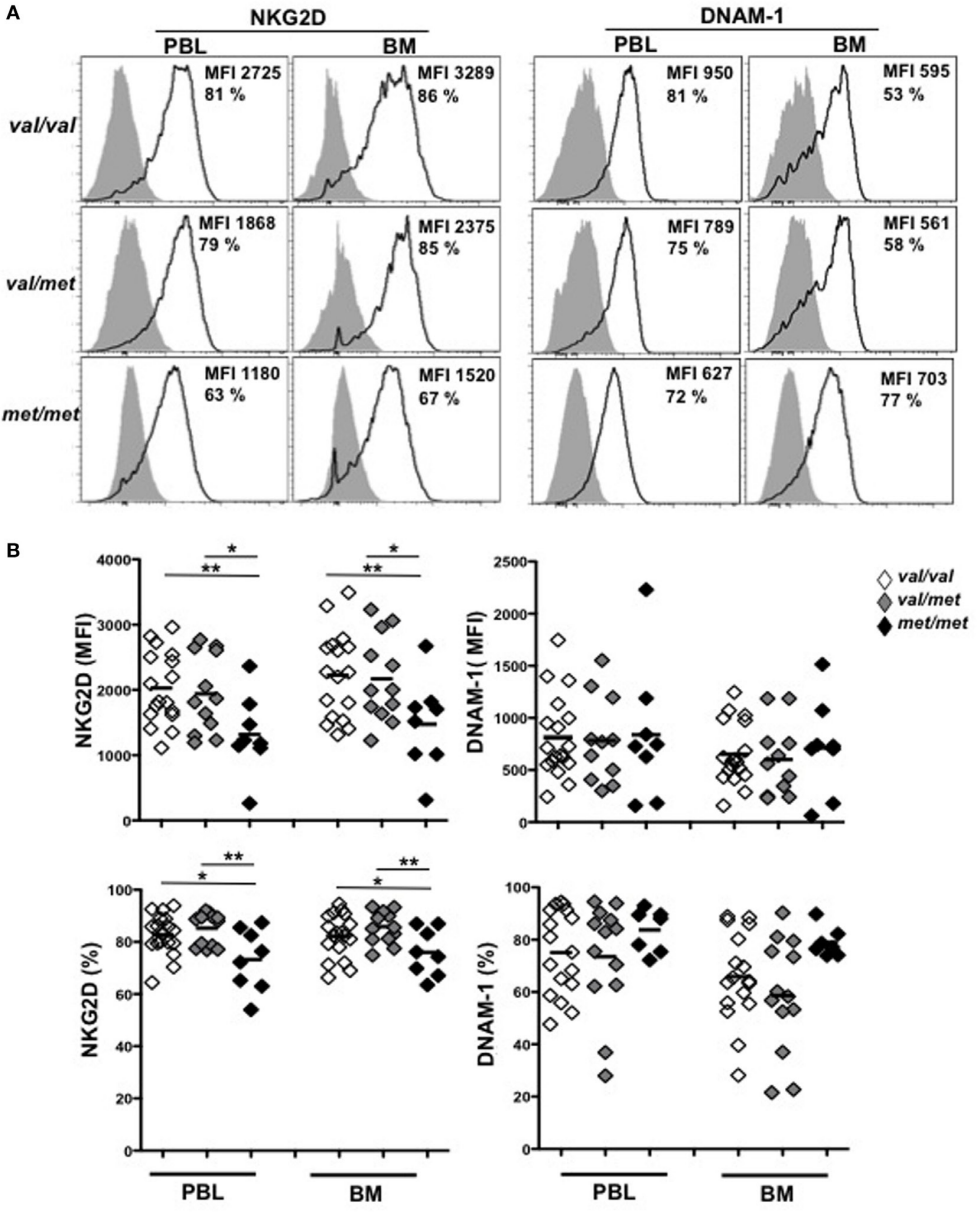
*MICA-129Met* allele is associated with an increased NKG2D downmodulation on natural killer (NK) cells gated from patients. Total cells derived from the bone marrow (BM) or the peripheral blood (PBL) derived from patients at different disease states (including smoldering, onset, and relapse) were stained with antibodies against CD45, CD138, CD56, and CD3. NKG2D or DNAM-1 expression was evaluated by gating on NK cells (CD45^+^CD138^−^CD3^−^ CD56^+^). **(A)** Representative histograms are shown. **(B)** Values reported represent the MFI values of NKG2D or DNAM-1 subtracted from the MFI value of the isotypic cIg or the percentage of NKG2D and DNAM-1. Total number of patients analyzed, *n* = 40 (Val/Val = 19, Val/Met *n* = 13, Met/Met = 8). Abbreviations: Val, valine; Met, methionine.

**Figure 5:**
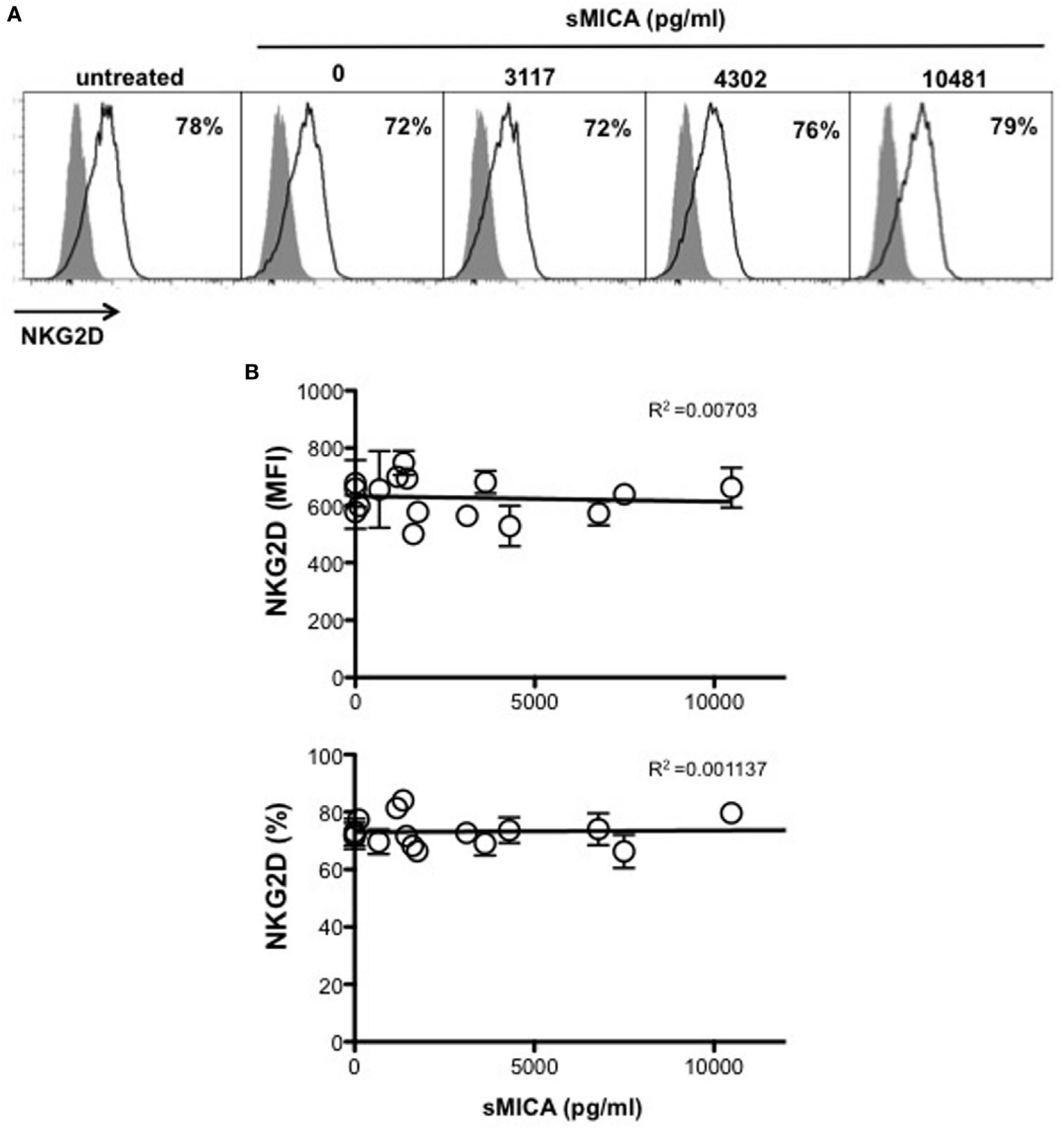
Soluble MICA levels in the serum of multiple myeloma (MM) patients carrying the Val allele do not correlate with change in NKG2D expression. Peripheral blood derived from healthy donors were incubated for 16 h with medium alone or serum derived from MGUS or MM patients at different disease states carrying at least one Val allele and containing variable levels of soluble MICA. Cells were harvested and NKG2D expression was evaluated by gating on CD3^−^CD56^+^ natural killer (NK) cells. **(A)** A representative experiment is shown. **(B)** Values reported on *y* axis represent the MFI value of NKG2D subtracted from the MFI value of the isotypic cIg (high panel) or the percentage of NKG2D positive cells (lower panel) and were correlated with soluble MICA levels of each patient as indicated on *x* axis. Total number of serum patients analyzed, *n* = 16 (3 MGUS, 6 smoldering, 6 onset, 1 relapse).

We next investigated whether MICA-129 polymorphism was also associated with different levels of NKG2D expression in MM patients. To this aim, NKG2D expression levels on *ex vivo* NK cells isolated from either PBL or BM of MM patients were evaluated. Cells were stained with anti-human CD45, -CD56, -CD3, -CD138 mAbs, along with mAbs specific for NKG2D and DNAM-1 activating receptors. After CD138 (corresponding to PCs) gate exclusion, analysis was performed on CD45^+^CD56^+^CD3^−^ total NK cells. Interestingly, our results demonstrate that NKG2D was significantly reduced on NK cells derived from *MICA-129Met/Met* patients compared to both *MICA-129Val/Val* and *MICA-129Val/Met* patients (Figures 4A,B). Indeed, the *MICA-129Met* allele that has a higher affinity to NKG2D, is able to induce significantly stronger downmodulation of NKG2D in both NK and CD8 T lymphocytes (6). Importantly, the expression levels of DNAM-1, used as control, were similar among all three different genotypes (Figures 4A,B), indicating that NKG2D downmodulation is an event likely associated to *MICA-129* dimorphism. Decreased NKG2D expression on *MICA-129Met/Met* patients was also observed on NK cell subsets expressing low and high levels of CD16 as shown in Figures S2A,B in Supplementary Material. These results suggest that NKG2D downmodulation in MM patients depends essentially on *MICA* genotype and it is not associated with soluble MICA levels. To further support this observation, sera containing different amounts of soluble MICA (derived from patients carrying at least one Val allele) were incubated with PBL derived from healthy donors and NKG2D expression was evaluated after 16 h by immunofluorescence and FACS analysis by gating on CD56^+^CD3^−^ NK cells. As shown in Figure 5, we did not observe a significant correlation between the levels of soluble MICA and the extent of NKG2D reduction.

### Residue Met129 Is Essential for Appropriate Positioning of the α_2–1_ Helix for NKG2D Recognition

**Figure 6:**
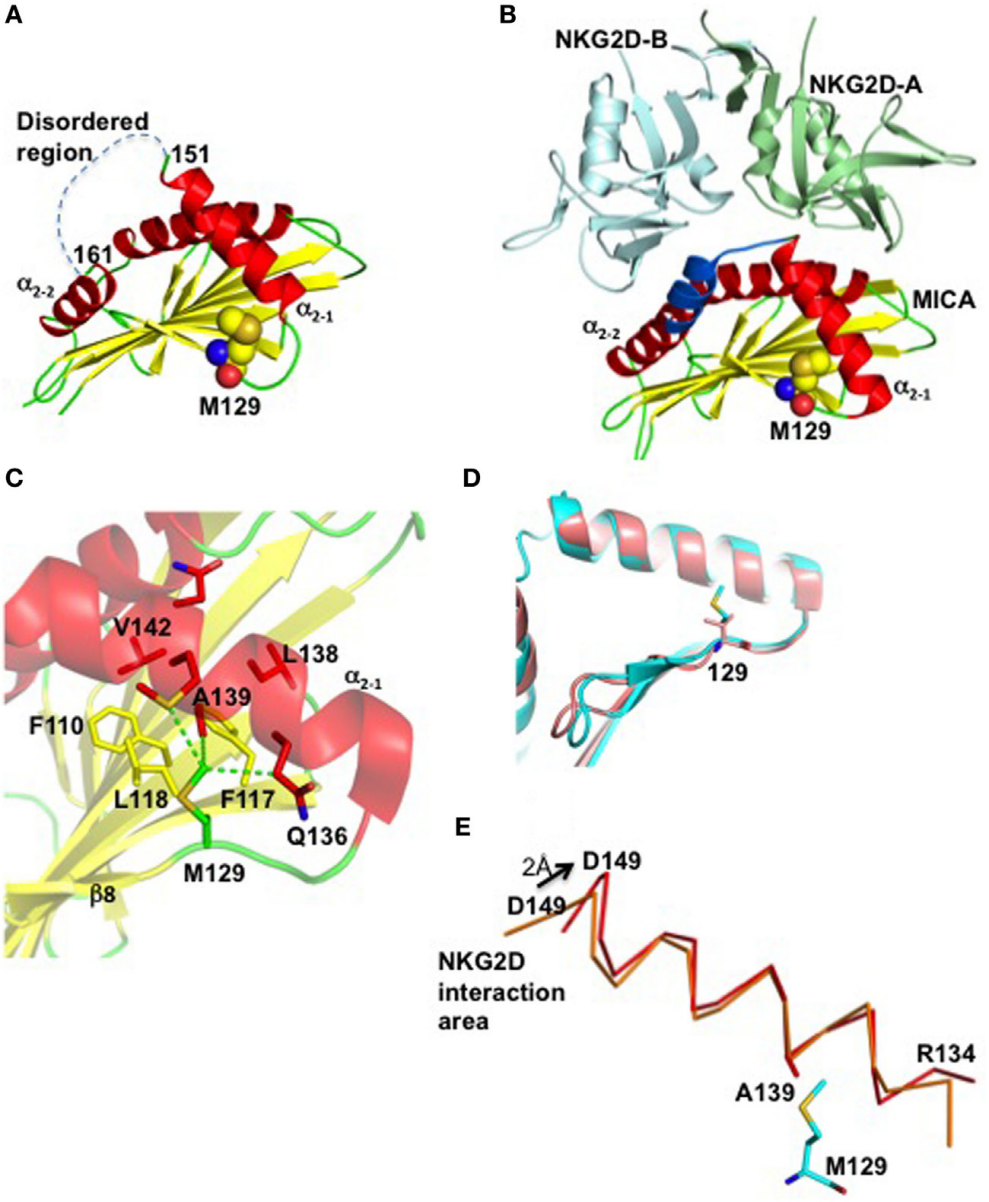
Residue Met129 is essential for appropriate positioning of the α_2–1_ helix for NKG2D recognition. **(A)** The crystal structure of the free form of MICA is colored according to its secondary structure, with helices, β-strands and loops in red, yellow, and green, respectively. Only the α1 and α2 regions of MICA are displayed. The region comprising residues 152–161 and which is not visible in the electron density due to disorder, is indicated by a dashed line. All atoms forming residue Met129 are indicated as balls with oxygen, nitrogen, sulfur, and carbon atoms colored in red, blue, orange, and yellow, respectively. **(B)** The crystal structure of the MICA/NKG2D complex reveals that the flexible 152–161 region (in blue) is stabilized through interactions with NKG2D. The two subunits of NKG2D receptor are displayed in green and cyan. Residue Met129 is localized far away from the MICA/NKG2D interface. **(C)** Residue Met129 plays a key role within a hydrophobic core formed between helix α_2–1_ and several β-sheet residues that surround and interact with Met129. The van der Waals interactions formed between Met129 and residues (in red) on the helix α_2–1_ are indicated by green dashed lines. **(D)** Superposition of the α1α2 domains of MICA and MICB, in pink and cyan, respectively, reveals that Met129 in MICA and Val129 in MICB take the same position and orientation, and demonstrates that MICA-129Met/Val dimorphism does not affect the conformation of the two MIC alleles (29). **(E)** The MICA α_2–1_ helix rotates following complex formation with NKG2D. Both ends of free MICA (red) are deviating from their position in the complex with NKG2D (orange), by 1.5 and 2.0 Å at the N- and C-termini, respectively.

Two crystal structures of MICA have been hitertho determined, one alone (26) and the other in complex with its receptor NKG2D (27). At first sight, MICA resembles classical MHC class I (MHC-I) molecules with three extracellular domains (α1, α2, and α3), a transmembrane segment that can vary significantly between different MICA alleles, and a carboxy-terminal cytoplasmic tail. However, in contrast to MHC-I, MICA does not bind to the β_2_microglobulin and does not present peptides in the cleft. Comparison of the two structures revealed that the receptor-free form of MICA is disordered within a section of the α_2_ region corresponding to residues 152 to 161, essential for NKG2D recognition (27) (Figure 6A). Interestingly, the crystal structure of MICA in complex with NKG2D revealed that these nine MICA residues, which link the helices α_2–1_ and α_2–2_, are ordered upon binding to NKG2D (Figure 6B). The crystal structures also revealed that residue Met129 is localized at the end of the small β8-strand, far away from the MICA/NKG2D interface. This residue forms, together with Trp127, Phe110, Phe117, and Leu118, a hydrophobic base on which the helix α_2–1_ docks (Figure 6C). The importance of such hydrophobic nucleus for the correct folding and orientation of a helix has been previously demonstrated (28). The hydrophobic residues Leu138, Ala139, Met140, and Val142 on the α_2–1_ helix face and interact with the β-sheet docking site described above (Figure 6C). The large and hydrophobic residue Met129 is at the heart of this putative nucleating site, forming van der Waals interactions with residues Gln136, Ala139, and Met140 all localized on the α_2–1_ helix (Figure 6C).

The clear differences in binding affinity between the two MICA variants and NKG2D have been previously suggested to be due to conformational changes (5). However, this is in our opinion unlike since comparison of the crystal structure of MICB (30) which comprises a Val residue at position 129, with the crystal structure of *MICA-129Met*, reveals the similarity of their conformations (Figure 6D). Instead, molecular modeling analysis indicated that replacement of residue Met129 with the much smaller Val residue removes at least three van der Waals interactions between the α_2–1_ helix and the β-sheet on which it docks, and generates a solvent accessible cavity in this hydrophobic core. Furthermore, comparative analysis of the two crystal structures of MICA indicates that the α_2–1_ helix rotates slightly upon NKG2D binding around the contact between Met129 and Ala139, resulting in significant movements at both ends of this helix (Figure 6E). Mutation of residue Met129, which we believe acts a lever stop for the helix α_2–1_ to build the conformation optimal for the NKG2D binding, would clearly have a significant impact on the direction and tilting of the helix. Thus, the Met to Val dimorphism could affect the MICA transition from disorder to form an optimal template for NKG2D recognition, providing a reasonable, although still hypothetical, explanation for the difference in the binding capacity of the two MICA variants to NKG2D.

## Discussion

In this study, we investigated the association of MICA genetic polymorphisms and sera levels with the progression of MM. Interestingly, our findings indicate that the *MICA-129Met/Val* dimorphism is associated with: (i) differential expression of both soluble and cell surface MICA, (ii) expression levels of NKG2D on *ex vivo* NK cells isolated from the BM and PBL of MM patients, and (iii) the disease state.

Polymorphisms of MICA have been largely investigated for their role in infections, autoimmune diseases, and cancer (31). Due to its functional consequences, a number of disease association studies with the *MICA-129* dimorphism have been previously performed (32). Interestingly, we found an higher frequency of relapse in MM patients carrying the *MICA-129Val/Val* genotype that was also observed by analyzing the patients outcome in response to the therapy.

Other studies in different cancer models have reported that the *MICA-129Val/Val* genotype can be associated with higher risk for nasopharyngeal (NC) and breast cancer (33, 34). Increasing evidence has pointed out a key role for the NKG2D activating receptor and its ligands in the surveillance of MM. In particular Rebmann and coworkers have shown that soluble MICA levels correlate with tumor progression, and this molecule has been proposed as a prognostic marker in MM (19). Our findings demonstrate that increased levels of soluble MICA can be found in MM patients sera during the progression from MGUS to relapse and clearly show for the first time that the presence of soluble MICA is associated with the *MICA-129Val* allele. As matter of fact, in other pathological conditions, including Ulcerative Colitis and Hepatitis B infection (35, 36), the *MICA-129Val/Val* genotype has been associated with the highest soluble MICA serum levels.

In line with our *in vivo* results showing both a higher expression of cell surface and soluble MICA in *MICA-129Val/Val* patients, Isernhagen and colleagues reported that soluble MICA levels as well as its cell-surface expression were higher in a panel of tumor and melanoma cell lines carrying the *MICA-129Val/Val* genotype (21). It is possible that the *MICA-129Val* allele has a higher transcriptional activity which might explain its effect on soluble and cell-surface MICA levels (37). Another non-excluding possibility is that the higher amount of soluble MICA-*129Val* could be directly due to the 30-times lower affinity of this variant to NKG2D compared to MICA-*129Met* resulting in a reduced transfer to NK cells and its accumulation on the surface of MM target cells.

Previous studies suggested that elevated levels of soluble MICA in the sera of cancer patients correlate with an increased NKG2D downregulation on PBL NK cells and T lymphocytes (22). However, it is still unclear whether soluble MICA has the capability to directly induce NKG2D downregulation and/or if additional soluble factors in the sera of cancer patients contribute to this effect. Infact, Paschen and coworkers demonstrated that elevated levels of soluble NKG2D ligands (i.e., MICA and ULBP2) in the sera of melanoma patients were not associated with a significant downregulation of NKG2D expression on peripheral NK cells (38). Even NK cells from rheumatoid arthritis patients with relatively high soluble MICA levels, did not show diminished NKG2D expression (39). Furthermore in MM, soluble MICA was not significantly associated with NKG2D downregulation and *in vitro* experiments with MM patients’ serum and culture supernatants, did not result in changes in NKG2D expression (40). Similarly, by incubating NK cells with sera from patients containing different amounts of soluble MICA, we did not find a significant correlation between the levels of soluble MICA and the extent of NKG2D downmodulation. Interestingly, we observed that the lowest levels of NKG2D on NK cells from MM patients, were associated with the *MICA-129Met/Met* genotype. In line with these findings, it has been shown that the *MICA-129Met* allele, with proven higher affinity to NKG2D, is able to induce significantly stronger downmodulation of NKG2D in both NK and CD8 T lymphocytes, and to better stimulate IFNγ production as compared to the *MICA-129Val* allele (6). It should be considered that receptor endocytosis not only leads to reduced cell-surface receptor abundance but also controls signaling outcome in NK cells as shown by Molfetta and coworkers who reported that ubiquitin-dependent NKG2D/DAP10 endocytosis was required for the activation of extracellular signal-regulated kinase and NK cell functions (41, 42). It is possible that cancer cells carrying the MICA-129*Met* allele could better induce NK cell activation that corresponded to a substantial NKG2D reduction observed in patients. By performing *in vitro* degranulation assays on NKG2D-sensitive targets, “*ex vivo*” unstimulated NK cells derived from patients had very low levels (below 5%) of degranulation, independently of the MICA genotype (data not shown). This result is in accordance with previous evidences indicating that stimulation of NKG2D alone is not sufficient to trigger cytotoxicity and/or cytokine production in resting human NK cells (43). In fact, effector functions mediated by this receptor rely on different factors including the activation status of NK cells, the cooperation with other NK activating receptors or with distinct cytokines (44). Thus, in MM patients, the tumor microenvironment, the cytokine milieu and the expression of other NK cell activating ligands on cancer cells can dictate the final outcome of the NKG2D-mediated NK cell response.

Our results obtained by structural modeling analysis suggested that the Met to Val dimorphism could affect the capacity of MICA to form an optimal template for NKG2D recognition. It is possible that the lowest NKG2D levels in MM patients with a MICA-*129Met/Met* genotype reflects the capacity of the *MICA-129Met* allele to more efficiently engage NKG2D and trigger NK cell activity in a cell–cell contact manner and this event appears to be independent from soluble MICA sera levels that are instead predominant in individuals carrying the *MICA-129Val* allele.

In conclusion, our data indicate that the *MICA-129Val* allele is associated with significantly higher levels of soluble MICA and an higher frequency of relapse and strongly suggest that the MICA genotype could be used as prognostic marker in alternative to soluble MICA if further data with higher numeric dimension will confirm these findings. Altogether, these observations could help to develop more personalized predictive biomarkers in MM.

## Materials and Methods

### Clinical Samples

Sera, PBMCs, and BM samples were obtained from MM patients enrolled at the Division of Hematology (Sapienza University of Rome). Informed and written consent in accordance with the Declaration of Helsinki was obtained from all patients, and approval was obtained from the Ethics Committee of the Sapienza University of Rome (Rif. 3373). Patients were classified according to the disease state. Patients (Onset and Relapse) were treated according to standard therapeutic protocols including the usage of VMP (Bortezomib, Melphalan, Prednisone), VD (Bortezomib, Dexamethasone), and RD (Lenalidomide, Dexamethasone).

### MICA Gene Typing

For the genotyping of MICA, genomic DNA derived from patients PBMCs was isolated from 1 × 10^6^ cells using the Genomic DNA purification kit according to the manufacturer’s instructions (Bioline, London, UK). Sequence-based typing of MICA was performed as described before (45). The sequence of a new MICA allele was identified and the name MICA*085 has been officially assigned by the WHO Nomenclature Committee for factors of the HLA System in February 2015 (Genbank accession: KP262025).

### Immunofluorescence and FACS Analysis

Analysis of MICA expression on patient-derived PCs was performed by gating the CD38^+^CD138^+^ PC population using the antibodies anti-MICA (clone 159227, R&D Systems, Minneapolis, MN, USA), anti-CD38/APC, and anti-CD138/FITC (both from BD Bioscience, San Jose, CA, USA) as previously reported (16); samples were acquired using a FACSCanto (BD Biosciences, San Jose, CA, USA) and a FACSCalibur (Becton Dickinson). Analysis of NKG2D and DNAM-1 on NK cells from PBMCs or BM aspirates was performed by gating on the CD45^+^CD138^−^CD3^−^CD56^+^ population using the antibodies anti-CD3/allophycocyanin-H7, anti-CD56/PE, anti-CD138/FITC, anti-CD45/PE-Cy7, anti-NKG2D/APC, or anti-DNAM-1/APC (BD Bioscience). In some experiments, anti-CD16/PerCP mAb was also used (BD Bioscience). In the experiments relative to Figure 4 and Figure S2 in Supplementary Material, all the patients-derived samples were acquired using a FACSCanto (BD Biosciences, San Jose, CA, USA). In the experiments relative to Figure 5, PBMCs derived from healthy donors were incubated with medium alone or serum derived from MGUS or MM patients for 16 h. After harvesting, cells were stained with antibodies from BD Bioscience: anti-CD3/PerCP, anti-CD56/APC, and anti-NKG2D/PE; samples were acquired using a FACSCalibur (Becton Dickinson).

Data analysis was performed using the FlowJo 9.3.2 program (TreeStar, Ashland, OR, USA).

### Enzyme-Linked Immunosorbent Assay (ELISA)

Enzyme-linked immunosorbent assays for soluble MICA, MICB, and ULBP1 were from R&D Systems (Minneapolis, MN, USA), and performed as previously described (46), with modifications (AMO1 anti-MICA capture mAb, 2 µg/ml, BAMOMAB, Germany) (47). Soluble ULBP2 was detected as previously described (15). Absorbance values of triplicate samples were obtained by subtracting readings at 540 nm from readings at 450 nm. Net absorbance was obtained by subtracting the reagent blank absorbance. Before the assay, sera samples were diluted in PBS/0.1% Triton X-100 (vol/vol) and incubated for 30 min at 37 C.

### Molecular Modeling of the MICA-Val129 Variant and Structural Analysis

In order to evaluate the structural consequences of the MICA polymorphism, we created a three-dimensional molecular model of the *MICA-Val129* variant, using the crystal structures of the free *MICA-Met129* molecule (PDB code 1B3J) and the *MICA-Met129/NKG2D* complex (PDB code 1HYR) (26, 27) as templates. The creation of the molecular model of the *MICA-Val129* variant, as well as all comparative structural analyses, was performed using the program Coot (48). Figure 5 was created using the program PyMol (PyMol Molecular Graphics System, Version 1.5.0.4 Schrödinger, LLC).

### Statistic

In all the experiments, statistic was performed using the unpaired Mann–Whitney test, except for Figure 1D in which the unpaired *t*-test with Welch’s correction was used. *<0.05; **<0.01; ***<0.001. χ^2^ test was used to analyze frequency data.

## Ethics Statement

Informed and written consent in accordance with the Declaration of Helsinki was obtained from all patients, and approval was obtained from the Ethics Committee of the Sapienza University of Rome.

## Author Contributions

FC, MG, and IN, extracted patients’ DNA, collected patients’ sera, and performed ELISA experiments. EV and AP performed experiments on bone marrow and peripheral blood of patients. DF, TSaribekyan, and JM performed MICA gene typing. AA and TSandalova performed structural modelling. EM, MP, and MR managed patients and evaluated clinical parameters. CF, ASoriani, MC, and CC, analyzed and discussed data. AZ and ASantoni designed the experiments and wrote the paper.

## Conflict of Interest Statement

The authors declare that the research was conducted in the absence of any commercial or financial relationships that could be construed as a potential conflict of interest.

## References

[1] Lanier LL. NKG2D receptor and its ligands in host defense. Cancer Immunol Res (2015) 3:575–82. 10.1158/2326-6066.CIR-15-0098 26041808 PMC4457299

[2] Cerboni C Zingoni A Cippitelli M Piccoli M Frati L Santoni A. Antigen-activated human T lymphocytes express cell-surface NKG2D ligands via an ATM/ATR-dependent mechanism and become susceptible to autologous NK-cell lysis. Blood (2007) 110:606–15. 10.1182/blood-2006-10-052720 17405908

[3] Nausch N Cerwenka A. NKG2D ligands in tumor immunity. Oncogene (2008) 27:5944–58. 10.1038/onc.2008.272 18836475

[4] Ashiru O Boutet P Fernandez-Messina L Aguera-Gonzalez S Skepper JN Vales-Gomez M Natural killer cell cytotoxicity is suppressed by exposure to the human NKG2D ligand MICA*008 that is shed by tumor cells in exosomes. Cancer Res (2010) 70:481–9. 10.1158/0008-5472.CAN-09-1688 20068167 PMC2817492

[5] Steinle A Li P Morris DL Groh V Lanier LL Strong RK Interactions of human NKG2D with its ligands MICA, MICB, and homologs of the mouse RAE-1 protein family. Immunogenetics (2001) 53:279–87. 10.1007/s002510100325 11491531

[6] Isernhagen A Malzahn D Viktorova E Elsner L Monecke S von Bonin F The MICA-129 dimorphism affects NKG2D signaling and outcome of hematopoietic stem cell transplantation. EMBO Mol Med (2015) 7:1480–502. 10.15252/emmm.201505246 26483398 PMC4644379

[7] Vallet S Pecherstorfer M Podar K. Adoptive cell therapy in multiple myeloma. Expert Opin Biol Ther (2017) 17:1511–22. 10.1080/14712598.2017.1375095 28857616

[8] Fionda C Soriani A Zingoni A Santoni A Cippitelli M. NKG2D and DNAM-1 ligands: molecular targets for NK cell-mediated immunotherapeutic intervention in multiple myeloma. Biomed Res Int (2015) 2015:178698. 10.1155/2015/178698 26161387 PMC4486747

[9] Boussi L Niesvizky R Advances in immunotherapy in multiple myeloma. Curr Opin Oncol (2017) 29:460–6. 10.1097/CCO.0000000000000407 28877078

[10] Pittari G Vago L Festuccia M Bonini C Mudawi D Giaccone L Restoring natural killer cell immunity against multiple myeloma in the era of new drugs. Front Immunol (2017) 8:1444. 10.3389/fimmu.2017.01444 29163516 PMC5682004

[11] Carbone E Neri P Mesuraca M Fulciniti MT Otsuki T Pende D HLA class I, NKG2D, and natural cytotoxicity receptors regulate multiple myeloma cell recognition by natural killer cells. Blood (2005) 105:251–8. 10.1182/blood-2004-04-1422 15328155

[12] Soriani A Zingoni A Cerboni C Iannitto ML Ricciardi MR Di Gialleonardo V ATM-ATR-dependent up-regulation of DNAM-1 and NKG2D ligands on multiple myeloma cells by therapeutic agents results in enhanced NK-cell susceptibility and is associated with a senescent phenotype. Blood (2009) 113:3503–11. 10.1182/blood-2008-08-173914 19098271

[13] Holdenrieder S Stieber P Peterfi A Nagel D Steinle A Salih HR. Soluble MICB in malignant diseases: analysis of diagnostic significance and correlation with soluble MICA. Cancer Immunol Immunother (2006) 55:1584–9. 10.1007/s00262-006-0167-1 16636811 PMC11030555

[14] Holdenrieder S Stieber P Peterfi A Nagel D Steinle A Salih HR. Soluble MICA in malignant diseases. Int J Cancer (2006) 118:684–7. 10.1002/ijc.21382 16094621

[15] Waldhauer I Steinle A. Proteolytic release of soluble UL16-binding protein 2 from tumor cells. Cancer Res (2006) 66:2520–6. 10.1158/0008-5472.CAN-05-2520 16510567

[16] Zingoni A Cecere F Vulpis E Fionda C Molfetta R Soriani A Genotoxic stress induces senescence-associated ADAM10-dependent release of NKG2D MIC ligands in multiple myeloma cells. J Immunol (2015) 195(2):736–48. 10.4049/jimmunol.1402643 26071561

[17] Zingoni A Vulpis E Nardone I Soriani A Fionda C Cippitelli M Targeting NKG2D and NKp30 ligands shedding to improve NK cell-based immunotherapy. Crit Rev Immunol (2016) 36:445–60. 10.1615/CritRevImmunol.2017020166 28845754

[18] McCann FE Eissmann P Onfelt B Leung R Davis DM. The activating NKG2D ligand MHC class I-related chain A transfers from target cells to NK cells in a manner that allows functional consequences. J Immunol (2007) 178:3418–26. 10.4049/jimmunol.178.6.3418 17339436

[19] Rebmann V Schutt P Brandhorst D Opalka B Moritz T Nowrousian MR Soluble MICA as an independent prognostic factor for the overall survival and progression-free survival of multiple myeloma patients. Clin Immunol (2007) 123:114–20. 10.1016/j.clim.2006.11.007 17218152

[20] Jinushi M Vanneman M Munshi NC Tai YT Prabhala RH Ritz J MHC class I chain-related protein A antibodies and shedding are associated with the progression of multiple myeloma. Proc Natl Acad Sci U S A (2008) 105:1285–90. 10.1073/pnas.0711293105 18202175 PMC2234130

[21] Isernhagen A Schilling D Monecke S Shah P Elsner L Walter L The MICA-129Met/Val dimorphism affects plasma membrane expression and shedding of the NKG2D ligand MICA. Immunogenetics (2015) 68:109–23. 10.1007/s00251-015-0884-8 26585323 PMC4728179

[22] Chitadze G Bhat J Lettau M Janssen O Kabelitz D. Generation of soluble NKG2D ligands: proteolytic cleavage, exosome secretion and functional implications. Scand J Immunol (2013) 78:120–9. 10.1111/sji.12072 23679194

[23] Fuerst D Neuchel C Niederwieser D Bunjes D Gramatzki M Wagner E Matching for the MICA-129 polymorphism is beneficial in unrelated hematopoietic stem cell transplantation. Blood (2016) 128:3169–76. 10.1182/blood-2016-05-716357 27811019

[24] Boukouaci W Busson M Peffault de Latour R Rocha V Suberbielle C Bengoufa D MICA-129 genotype, soluble MICA, and anti-MICA antibodies as biomarkers of chronic graft-versus-host disease. Blood (2009) 114:5216–24. 10.1182/blood-2009-04-217430 19786616

[25] Lopez-Hernandez R Valdes M Lucas D Campillo JA Martinez-Garcia P Salama H Association analysis of MICA gene polymorphism and MICA-129 dimorphism with inflammatory bowel disease susceptibility in a Spanish population. Hum Immunol (2010) 71:512–4. 10.1016/j.humimm.2010.02.003 20152875

[26] Li P Willie ST Bauer S Morris DL Spies T Strong RK. Crystal structure of the MHC class I homolog MIC-A, a gammadelta T cell ligand. Immunity (1999) 10:577–84. 10.1016/S1074-7613(00)80057-6 10367903

[27] Li P Morris DL Willcox BE Steinle A Spies T Strong RK. Complex structure of the activating immunoreceptor NKG2D and its MHC class I-like ligand MICA. Nat Immunol (2001) 2:443–51. 10.1038/87757 11323699

[28] Wensley BG Kwa LG Shammas SL Rogers JM Clarke J. Protein folding: adding a nucleus to guide helix docking reduces landscape roughness. J Mol Biol (2012) 423:273–83. 10.1016/j.jmb.2012.08.003 22917971 PMC3469821

[29] Muller S Zocher G Steinle A Stehle T. Structure of the HCMV UL16-MICB complex elucidates select binding of a viral immunoevasin to diverse NKG2D ligands. PLoS Pathog (2010) 6:e1000723. 10.1371/journal.ppat.1000723 20090832 PMC2797645

[30] Holmes MA Li P Petersdorf EW Strong RK. Structural studies of allelic diversity of the MHC class I homolog MIC-B, a stress-inducible ligand for the activating immunoreceptor NKG2D. J Immunol (2002) 169:1395–400. 10.4049/jimmunol.169.3.1395 12133964

[31] Chen D Gyllensten U MICA polymorphism: biology and importance in cancer. Carcinogenesis (2014) 35:2633–42. 10.1093/carcin/bgu215 25330802

[32] Isernhagen A Malzahn D Bickeboller H Dressel R. Impact of the MICA-129Met/Val dimorphism on NKG2D-mediated biological functions and disease risks. Front Immunol (2016) 7:588. 10.3389/fimmu.2016.00588 28018354 PMC5149524

[33] Douik H Ben Chaaben A Attia Romdhane N Romdhane HB Mamoghli T Fortier C Association of MICA-129 polymorphism with nasopharyngeal cancer risk in a Tunisian population. Hum Immunol (2009) 70:45–8. 10.1016/j.humimm.2008.10.008 19000729

[34] Ouni N Ben Chaaben A Kablouti G Lajnef M Ayari F Abaza H MICA-129Met/Val polymorphism is associated with early-onset breast cancer risk. Immunol Invest (2017) 46(6):603–14. 10.1080/08820139.2017.1336175 28742417

[35] Tong HV Toan NL Song LH Bock CT Kremsner PG Velavan TP. Hepatitis B virus-induced hepatocellular carcinoma: functional roles of MICA variants. J Viral Hepat (2013) 20:687–98. 10.1111/jvh.12089 24010643

[36] Zhao J Jiang Y Lei Y Zou K Wang C Huang S Functional MICA-129 polymorphism and serum levels of soluble MICA are correlated with ulcerative colitis in Chinese patients. J Gastroenterol Hepatol (2011) 26:593–8. 10.1111/j.1440-1746.2010.06524.x 21155878

[37] Lo PH Urabe Y Kumar V Tanikawa C Koike K Kato N Identification of a functional variant in the MICA promoter which regulates MICA expression and increases HCV-related hepatocellular carcinoma risk. PLoS One (2013) 8:e61279. 10.1371/journal.pone.0061279 23593449 PMC3623965

[38] Paschen A Sucker A Hill B Moll I Zapatka M Nguyen XD Differential clinical significance of individual NKG2D ligands in melanoma: soluble ULBP2 as an indicator of poor prognosis superior to S100B. Clin Cancer Res (2009) 15:5208–15. 10.1158/1078-0432.CCR-09-0886 19671853

[39] Groh V Bruhl A El-Gabalawy H Nelson JL Spies T. Stimulation of T cell autoreactivity by anomalous expression of NKG2D and its MIC ligands in rheumatoid arthritis. Proc Natl Acad Sci U S A (2003) 100:9452–7. 10.1073/pnas.1632807100 12878725 PMC170939

[40] von Lilienfeld-Toal M Frank S Leyendecker C Feyler S Jarmin S Morgan R Reduced immune effector cell NKG2D expression and increased levels of soluble NKG2D ligands in multiple myeloma may not be causally linked. Cancer Immunol Immunother (2010) 59:829–39. 10.1007/s00262-009-0807-3 20024547 PMC11030819

[41] Quatrini L Molfetta R Zitti B Peruzzi G Fionda C Capuano C Ubiquitin-dependent endocytosis of NKG2D-DAP10 receptor complexes activates signaling and functions in human NK cells. Sci Signal (2015) 8:ra108. 10.1126/scisignal.aab2724 26508790

[42] Molfetta R Quatrini L Zitti B Capuano C Galandrini R Santoni A Regulation of NKG2D expression and signaling by endocytosis. Trends Immunol (2016) 37:790–802. 10.1016/j.it.2016.08.015 27667711

[43] Bryceson YT March ME Ljunggren HG Long EO. Synergy among receptors on resting NK cells for the activation of natural cytotoxicity and cytokine secretion. Blood (2006) 107:159–66. 10.1182/blood-2005-04-1351 16150947 PMC1895346

[44] Bryceson YT March ME Ljunggren HG Long EO. Activation, coactivation, and costimulation of resting human natural killer cells. Immunol Rev (2006) 214:73–91. 10.1111/j.1600-065X.2006.00457.x 17100877 PMC3845883

[45] Furst D Solgi G Recker K Mytilineos D Schrezenmeier H Mytilineos J. Sequence-based typing of major histocompatibility complex class I chain-related gene A alleles by use of exons 2-5 information. Tissue Antigens (2011) 77:201–5. 10.1111/j.1399-0039.2010.01601.x 21299524

[46] Cerboni C Ardolino M Santoni A Zingoni A. Detuning CD8+ T lymphocytes by down-regulation of the activating receptor NKG2D: role of NKG2D ligands released by activated T cells. Blood (2009) 113:2955–64. 10.1182/blood-2008-06-165944 19124832

[47] Cox MC Battella S La Scaleia R Pelliccia S Di Napoli A Porzia A Tumor-associated and immunochemotherapy-dependent long-term alterations of the peripheral blood NK cell compartment in DLBCL patients. Oncoimmunology (2015) 4:e990773. 10.4161/2162402X.2014.990773 25949906 PMC4404844

[48] Emsley P Lohkamp B Scott WG Cowtan K. Features and development of Coot. Acta Crystallogr D Biol Crystallogr (2010) 66:486–501. 10.1107/S0907444910007493 20383002 PMC2852313

