# Supplementary material for: MICA-129 Dimorphism and Soluble MICA Are Associated With the Progression of Multiple Myeloma
Source: Front Immunol. 2018 May 1;9:926. doi: 10.3389/fimmu.2018.00926 (PMC5938351; doi:10.3389/fimmu.2018.00926)
Supplement: Supplementary file 1 [file Image_1.PDF]

A

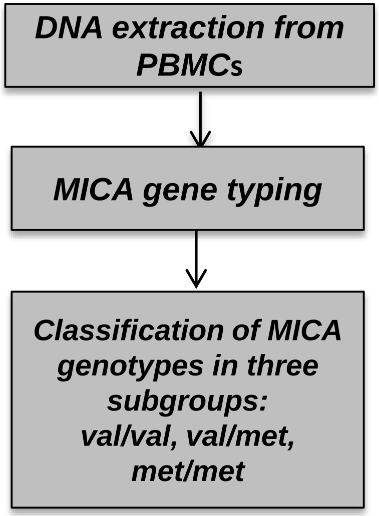

B

MICA129 genotypes	%
val/val	36 (n=49)
val/met	42 (n=57)
met/met	22 (n=31)

C

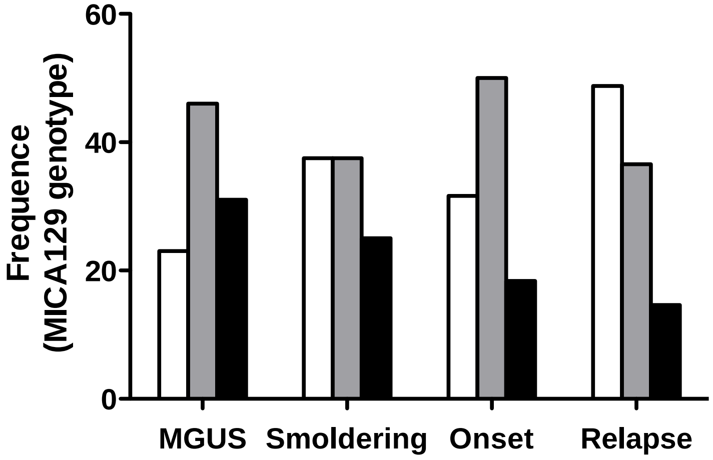

Supplementary figure 1. Frequence of MICA-129 polymorphisms in the cohort of MGUS and MM patients. A) Schematic representation of the procedure used to perform the MICA gene typing. **B)** Frequencies of MICA genotypes in patients classified based on the met and val substitution at position 129 of the a heavy chain of the MICA extracellular domain. *Val-129* alleles: MICA*004, *005, *006, *008, *009, *010, *016, *019, *027, *085. *Met-129* alleles: MICA*001, *002, *007, *011, *012, *015 . *017, *018, *021, **047. Total number of patients: 137. **C)** Frequence of distinct MICA genotypes across the same disease status.
