# Supplementary material for: MICA-129 Dimorphism and Soluble MICA Are Associated With the Progression of Multiple Myeloma
Source: Front Immunol. 2018 May 1;9:926. doi: 10.3389/fimmu.2018.00926 (PMC5938351; doi:10.3389/fimmu.2018.00926)
Supplement: Supplementary file 2 [file Image_2.PDF]

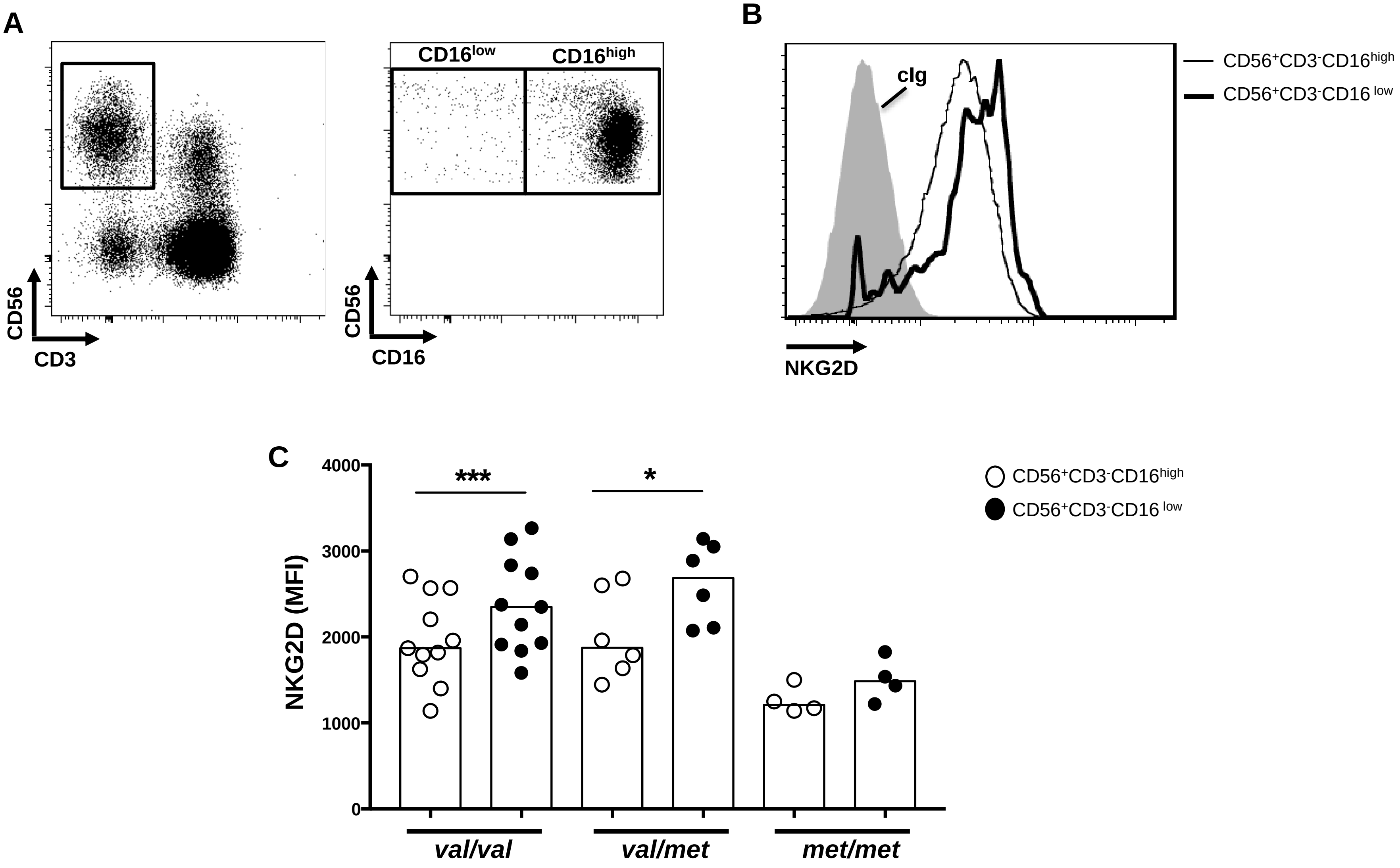

Supplementary figure 2. Evaluation of NKG2D expression on CD16^{low} and CD16^{high} NK cell subsets. PBMCs derived from MM patients were stained with a mixture of antibodies containing anti-CD3, anti-CD16, anti-CD56 and anti-NKG2D. (A) Gating strategy evaluating NKG2D expression on CD56+CD3-CD16^{low} and CD56+CD3-CD16^{high} NK cell subsets. (B) Representative histogram showing NKG2D expression on CD56+CD3-CD16^{low} and CD56+CD3-CD16^{high} NK cell subsets. (C) Data collected from different patients are shown. Total number of patients: 21; (val/val, n=11; val/met, n=6; met/met, n=4). Statistical differences are shown.
